# Traumatic dental injuries among 12-year-old Jordanian schoolchildren: an investigation on obesity and other risk factors

**DOI:** 10.1186/1472-6831-14-101

**Published:** 2014-08-07

**Authors:** Tala Tariq Al-Bajjali, Lamis Darwish Rajab

**Affiliations:** 1 Faculty of Dentistry, Department of Pediatric Dentistry, University of Jordan, Amman, P.O. Box 13595, Jordan

**Keywords:** Traumatic dental injuries, Obesity, Risk factors

## Abstract

**Background:**

Traumatic dental injury (TDI) is an important public dental health problem among schoolchildren. The aim of the study was to investigate the relationship between TDIs, obesity, and other possible associated factors like gender, overjet, lip coverage, parents’ education level and family income among 12-year old Jordanian schoolchildren.

**Methods:**

A cross-sectional population-based study examined a total of 1015 schoolchildren attending 34 schools randomly selected from urban and rural areas of Amman-the capital city of Jordan. The epidemiological classification adopted by WHO and modified by Andreasen et al. was used to classify TDIs. Obesity was defined according to the international cut-off points of body mass index for boys and girls between 2 and 20 years old.

**Results:**

The prevalence of TDIs was 16.3%. Enamel fracture was the most common type of TDIs (65%). Neither parents’ education level nor family income had a significant effect on TDI occurrence (P > 0.05). Results of multiple logistic regression showed that TDIs were significantly more prevalent among males (OR = 1.42, CI; 1.01-2.01, P < 0.05), and children with inadequate lip coverage (OR = 1.95, CI; 1.35-2.81, P < 0.05). The relationship between TDIs and obesity was not statistically significant (P > 0.05).

**Conclusions:**

Being a male with inadequate lip coverage was associated with higher probability of having a TDI. Obesity had no significant effect on the occurrence of TDIs.

## Background

Traumatic dental injuries (TDIs) can cause an irreversible dental loss with inconvenient consequences to the child and parents because of the long term follow-up and possible complications that can occur even after years post treatment. The economic costs of treatment, its impact on the oral health-related quality of life and the possibility of prevention, have made TDI a serious public dental health problem in children [1-4]. According to recent population-based studies, the prevalence of TDIs to permanent anterior teeth is high worldwide ranging from 4.1% to 58.6% [1,2]. Oral factors (increased overjet with protrusion, incompetent lips), environmental determinant, and human behavior were found to increase the risk for TDIs [1]. Childhood obesity has become a global epidemic recently, and it has been pointed out in the literature as another risk factor to TDIs [1,3-5]. The relationship between obesity and TDIs has been investigated in few studies (Table 1) [3-8]. Nevertheless, the possible relationship between obesity and TDIs is still unclear. While two studies considered obesity a risk factor for TDIs [3,4], other studies showed no statistically significant relationship [6-8]. In Jordan, few epidemiological studies are available on TDIs to anterior permanent teeth among schoolchildren [9-11], but none of them has explored the relationship between TDIs and obesity. In a study comparing the distribution of TDIs among 10-12-year-old children between urban and rural areas in Jordan, Hamdan and Rock [9] found a prevalence of 19.1% and 15.5%, respectively. In another cross-sectional study among 1.878 12-year-old Jordanian schoolchildren, Hamdan and Rajab [10] found a prevalence of 13.8% and a very high treatment need. However, a more recent national survey [11] reported a lower prevalence of TDIs (5.5%) among 12-year-old schoolchildren but great treatment neglect.

**Table 1:** Studies on the relationship between obesity and traumatic dental injuries (TDIs)

**Author**	**Country**	**N**	**Ages in years**	**Conclusions**
Petti et al. [3]	Italy	938	6-11	- Obesity significantly increased the risk of TDIs. One-third of obese children was affected vs. only one fifth of other subjects.
Nicolau et al. [4]	Brazil	652	13	- Being from a non-nuclear family, overweight and a boy increased the risk of having a TDI.
				- Overweight children were 1.93 times more likely to have dental injuries than those who were not overweight
Granville-Garcia et al. [5]	Brazil	2651	1-5	-Overweight/obese children had 2.5 times more trauma than non-overweight/obese ones
Soriano et al. [6]	Brazil	1046	12	- Obese subjects sustained more TDIs than non-obese subjects. However, it was concluded that the presence of obesity was not associated to TDIs in adolescents from Recife, Brazil.
Ărtun and Al-Azemi [7]	Kuwait	1583	13-14	- No difference was detected in TDIs rate among the subjects in the three BMI categories
Damé-Teixeira et al. [8]	Brazil	1528	12	- No significant association was found between BMI and obesity.

The aim of this study was to investigate the relationship between TDIs and obesity among 12-year-old Jordanian schoolchildren along with other possible associated factors such as gender, overjet, lip coverage, parents’ education level and family income.

## Methods

### Ethical approval

Before the commencement of the study, the research protocol was approved by the Institutional Review Board (IRB) at the University of Jordan. Permission from the Ministry of Education was also obtained for the examination of schoolchildren. Formal letters were sent to the selected schools attached with the required authorities’ permission. All parents or legal guardians were asked to sign a written informed consent form authorizing the enrollment of their children in the project, through which, the aims and importance of the study were explained.

### The study design and population

A cross-sectional survey was carried out with the target population comprising children aged 12 years regularly attending private, public, the United Nation Relief and Work Agency (UNRWA) schools in Amman- the capital of Jordan, and neighboring regions of the middle Badia. A stratified two-stage random cluster sample design was applied, using schools as the primary sampling unit.

### Sample size and sampling procedure

According to the sample size equation, a minimum sample size of 183 children was required to achieve a level of precision with a standard error of 5%. A 95% confidence interval level and a 13.8% prevalence of TDIs (the dependent variable) reported in a previous study carried out in Amman [10], were used for the calculation of the sample size. The required sample size was increased to avoid Type II sampling error, and to decrease the effect of confounding variables and increase the precision of the study. In Jordan which is a small developing country with limited economic resources, the survey was an opportunity to provide dental services to the maximum number of school children who were willing to participate.

The lists of schools, their address, phone numbers, number of classes and total number of children in the 6^th^ grade were obtained from the Department of Statistics/Ministry of Education. According to official figures, there were 48494 children attending the 6^th^ grade in 960 schools in Amman, and neighboring regions of the middle Badia. Using a random sample generator on an electronic website [12], a cluster random sample was selected. Taking proportionality of the number of students between different school types into consideration, the final sample size of this survey was increased to include 1025 children, 260 from private schools, 360 from public schools, and 210 from UNRWA schools in Amman. In addition, 195 children were recruited from public schools in middle Badia, a rural area with only public schools.

### The questionnaire

A previously tested short questionnaire about socioeconomic indicators was included in the consent form with questions about parent’s educational level and family income to be answered by parents who agreed their children participation.

### Diagnostic criteria of traumatic dental injuries

The study sample included 12-year-old schoolchildren, who gave consent forms from parents/legal guardian on the day of interview and examination. Children with medical condition that would affect growth, and consequently anthropometric parameters, and those undergoing fixed orthodontic treatment were excluded from the study.

TDIs were diagnosed by clinical examination. Radiographic examination and pulp vitality test were not used for the diagnosis of TDI. The study was limited to anterior permanent incisor teeth as other teeth are seldom affected [2,10,13,14]. The WHO epidemiological classification of TDIs modified by Andreasen et al. [15] was adopted to record injuries. Types of treatment needed and provided criteria were also recorded according to previously adopted protocols [14,16].

### Examiner reliability

All clinical examinations were carried out on patients by a postgraduate student (TTA) who was trained and calibrated by a University Professor of Pediatric Dentistry (LDR), for anthropometric measurement, oral examination, and the criteria used to identify dental injuries before the commencement of the study. There was 98.3% agreement during calibration. During the examination process, intra-examiner reliability was checked through duplicate examination of every 10^th^ subject. Consequently, 50 children were examined twice and a high intra-examiner kappa value of 92.3 was obtained indicating a very good agreement.

### Interview

All children were interviewed by the researcher (TTA) for participation in sports, and whether or not they use a mouthguard. Only those who had clinical evidence of TDIs were interviewed regarding details of the injury event including when, where, and how the injury occurred.

### Oral examination

Lip coverage was recorded according to the criteria adopted by Burden [17]. If the lip covered the upper incisors during the rest position, lip coverage was rated as adequate. If the greater part of the upper incisors was exposed or lip strain was evident upon closure, lip coverage was rated as inadequate [17]. Incisal overjet was measured in millimeters, from the labial surface of the mandibular incisors to the incisal edge of the most prominent maxillary incisor, with disposable ruler being held parallel to the occlusal plane and radial to the arch [17]. The number of traumatized teeth per child was also documented. Dental examination was conducted using sterilized dental mirrors. Probes were used to detect composite restorations. Children were seated and examined in a room with good natural lighting supplemented with a portable head light. The universal infection control precautions were followed during examination, and gauzes were used to dry teeth and remove any residual debris when necessary.

### Anthropometric measurements

Weight and height were measured for each subject according to standard methods adopted by Center for Disease Control and Prevention (CDC) [18,19]. Weight was measured in kilograms (Kg) using digital scaler and height was measured in centimeters (cm) using a meter setup measure fixed to the wall. BMI was calculated during data analysis according to the standard formula [18]. Overweight and obesity were assessed after referring to the CDC growth charts specific for age and sex. Boys with BMI equal or above 24.2 were considered obese while girls with BMI equal or above 25.2 were considered obese.

### Data analysis

Data were processed and analyzed using the Statistical Package for Social Sciences, version 17 (SPSS Inc., Chicago, IL, USA). Statistical analysis of association of TDIs (the dependent variable) with different independent variables was performed using Chi-square procedures. Logistic regression was used for assessment of potential predictors of TDIs. Odds ratio were also calculated with 95% confidence intervals and adjusted for significantly associated variables to identify the independent contribution of each variable and avoid any possible confounding effect. The inclusion criterion of the independent variables to enter the model was set at 0.05. The Statistical significance was set at ≤ 0.05.

## Results

From 1780 distributed consent forms, 1025 were returned by schoolchildren with a response rate of 57.6%. Ten children were excluded (nine children with fixed orthodontic treatment and one with medical condition that affects bone, and consequently BMI values). A total of 1015 sixth grade schoolchildren were examined. There were 545 (53.7%) males and 460 (46.3%) females. One hundred sixty five children experienced TDIs. The overall prevalence of TDIs was 16.3% among 12-year old Jordanian schoolchildren. Table 2 presents the distribution of different types of TDIs among injured teeth.

**Table 2:** Types of injury according to the epidemiological classification adopted by WHO and modified by Andreasen et al. (2007)

**WHO codes**	**Type of injury**	**N**	**%**
0	No sign of injury	1100	83.3
1	Treated injury	14	1.0
2	Enamel fracture only	143	10.8
3	Enamel/Dentin fracture	44	3.4
4	Pulp involvement	17	1.3
5	Missing teeth due to trauma	2	0.2
	Total	1320	100.0

There were 220 traumatized teeth. One hundred fourteen children experienced TDI in one tooth and 48 children in two teeth. Upper central incisors were the most common affected teeth (92.7%), with the left side slightly more involved than the right side. Enamel fracture was the most common type of injuries (65%) followed by enamel-dentin fracture (20%). The majority of injured teeth were untreated (93.6%). Only 14 teeth (6.4%) were treated mainly with simple composite restorations. The most common season in which injuries occurred was summer (34.5%), and the least common season was spring (2.4%). The most common place for injury was home (30.9%), followed by street (13.3%). The leading cause of injury was fall (30.9%) followed by collision (17.1%).

Prevalence of TDIs along with frequency distributions of demographic characteristics, socio-economic indicators, and physical characteristics of the sample were presented in Table 3. Males had significantly higher prevalence of TDIs than females (18.9% vs. 13.5%). Schoolchildren from rural areas had higher prevalence of TDIs (19.1%) compared to those from urban areas (15.6%). However, the difference was not statistically significant (P > 0.05). Children in private schools had the highest prevalence of TDIs (16.4%). The difference in TDI occurrence between school types was not statistically significant (P > 0.05). Neither parents’ education level nor family income had a significant effect on TDI occurrence (P > 0.05). Almost two thirds of the sample were practicing sports regularly, with only six schoolchildren reported using a mouthguard mostly during taekwondo training.

**Table 3:** Prevalence of traumatic dental injuries (TDIs) among 12-year-old Jordanian schoolchildren (N = 1015) by demographic characteristics, socio-economic indicators, and physical characteristics

**Variable**	**TDI**	**No TDI**	**Total**	**P-value**
	**N (%)**	**N (%)**	**N (%)**	
Gender*				
Male	103 (18.9)	442 (81.1)	545 (53.7)	P = 0.01
Female	62 (13.2)	408 (86.8)	470 (46.3)	
*School area*				
Urban	128 (15.6)	693 (84.4)	821 (80.9)	P = 0.24
Rural	37 (19.1)	157 (80.9)	194 (19.1)	
*School type*				
Private	41 (16.4)	209 (83.6)	250 (24.6)	P = 0.90
Public	92 (16.6)	463 (83.4)	555 (54.7)	
UNRWA	32 (15.2)	178 (84.8)	210 (20.7)	
*Father's education level*				
High school or less	90 (16.3)	472 (83.7)	562 (55.4)	P = 0.96
Diploma, Bachelor	63 (16.4)	321 (83.6)	384 (37.9)	
Post graduate degree	12 (17.4)	57 (82.6)	69 (6.8)	
*Mother's education level*				
High school or less	103 (16.1)	535 (83.9)	638 (62.9)	P = 0.37
Diploma, Bachelor	54 (16.2)	280 (83.8)	334 (32.9)	
Post graduate degree	8 (18.6)	35 (81.4)	43 (4.2)	
*Family income*				
Less than enough	52 (16.1)	270 (83.9)	322 (31.7)	P = 0.64
Enough	110 (16.7)	548 (83.3)	658 (64.8)	
More than enough	1 (6.7)	14 (93.3)	15 (1.5)	
No answer	2 (10.0)	18 (90.0)	20 (2.0)	
Overjet*				
≤ 3 mm	85 (14.2)	513 (85.8)	598 (58.9)	P = 0.04
>3 mm	80 (19.2)	337 (80.8)	417 (41.1)	
Lip coverage**				
Adequate	51 (11.0)	413 (89.0)	464 (45.7)	P = 0.00
Inadequate	114 (20.7)	437 (79.3)	551 (54.3)	
*BMI*				
Obese	12 (10.7)	100 ( 89.3)	112 (11.0)	P = 0.09
Non-obese	153 (16.9)	750 ( 83.1)	903 (89.0)	
*Total*	165 (16.3)	850 (83.7)	1015 (100)	

*Statistically significant at P < 0.05, **statistically significant at P < 0.001.

Forty one percent of schoolchildren had increased overjet beyond 3 mm and 54.3% had inadequate lip coverage. Having an increased overjet was significantly associated with TDIs (P < 0.05). Children with inadequate lip coverage had significantly higher prevalence of TDIs (20.7%) comparing to other children (11.0%).

According to CDC growth charts; of the total 1015 children, 16.3% were overweight (14.7% of males and 17.9% of females) and 11% were obese (12.7% of males and 9.1% of females). TDIs occurred more frequently in non-obese children when compared to obese ones (16.9% vs.10.7%). Moreover, underweight children had the highest prevalence (20.0%) of TDIs followed by healthy weight and overweight children; 17.2% and 15.9%, respectively. However, the association was not statistically significant (P = 0.09).

Logistic regression analysis with a stepwise selection procedure was used to investigate the simultaneous influence of different independent variables (gender, overjet, and lip coverage) that were significantly associated to TDI, the dependent variable, as shown in Table 4.

**Table 4:** Logistic regression analysis of variables related to traumatic dental injuries

	**TDIs N (%)**		**Unadjusted OR**	**P**	**Adjusted OR**	**P**
	**Yes**	**No**	**(95% CI)**		**(95% CI)**	
*Gender*						
Male	103 (18.9)	442 (81.1)	1.53 (1.09-2.16)	0.01	1.42 (1.01-2.01)	0.05
Female	62 (13.2)	408 (86.8)	**-**			
*Overjet*						
*≤ 3 mm*	85 (14.2)	513 (85.8)	**-**	0.04	1.22 (0.86-1.72)	0.27
*>3 mm*	80 (19.2)	337 (80.8)	1.43 (1.03-2.00)			
Lip coverage						
Adequate	51 (11.0)	413 (89.0)	**-**	0.00	1.95 (1.35-2.81)	0.00
Inadequate	114 (20.7)	437 (79.3)	2.11 (1.48-3.02)			

Children with inadequate lip coverage were about two times more likely to be exposed to dental trauma than other children (OR = 1.95, 95% CI: 1.35-2.81). The odds of having TDI in males were about 1.4 times more compared to females (OR = 1.42, 95% CI: 1.01-2.01). According to the logistic regression model, overjet did not show any significant association with TDIs.

## Discussion

The present study is the first population-based epidemiological survey relating TDIs to obesity among 12-year-old schoolchildren living in Jordan. The value of this population-based survey was in investigating two major common public health problems found in children. Exploring the association of obesity, together with other possible associated factors, and TDIs was important to avoid bias arising from other confounding variables. However, low response rate due to poor compliance of children and their parents presented an important limitation of the study and attributed to the self-selection bias commonly found in voluntary surveys. Assessing treated and untreated TDIs added an advantage to this survey that is not found in hospital-based and clinical-based studies resulting in a more accurate presentation of TDIs’ prevalence in the population studied. The prevalence of TDIs obtained using the WHO classification can be directly compared to those recorded in other epidemiological surveys carried out in different countries [2]. Due to the lack of diagnostic aids such as radiographs; root fractures and luxation injuries were overlooked making the prevalence of TDIs underestimated. However, tooth discoloration was included in the ‘pulp involvement’ category of TDIs. Recording the presence of treated and untreated TDIs, and the types of treatment provided and treatment needed, was of great significance to address the urgent need for a public program to increase the awareness of parents and school teachers on the importance of TDI management.

A prevalence of 16.3% of TDIs was identified. This prevalence is lower than 19.2% found in a previous survey conducted in Jordan in 1995 [9], but higher when compared to 13.8% reported in a population-based study among similar age group in Jordan in 2003 [10], and 5.5% reported in 2013 [11]. It was similar to that found in a study carried out in Palestinian towns (17.7%) [14], as well as in other studies conducted previously in Brazil [4,20,21]. Lower prevalences ranging from 11.5% to14.7% were reported in other Middle Eastern countries [7,22,23]. The difference in the prevalence of TDIs can be explained by different methodology, diagnostic criteria, populations, and also geographic and cultural variations in the studied populations that influence the type of activities that children usually practice at home, in school and outdoor.

The most frequent injury was enamel fractures (65%) followed by fractures involving enamel and dentin (20%), this corroborates the findings of previous studies in several countries [2,9,21,23-25]. This finding explains the high prevalence of TDIs found in this survey compared to that found in other hospital-based studies where most enamel fractures are left untreated and are not recorded [26,27]. Other authors reported enamel-dentin fracture as the most frequent injury to permanent incisors [10,11,14]. The upper central incisors were the most injured teeth (92.7%), which is similar to the findings of previous studies in Jordan and other countries [10,11,14,26,27]. This is because the morphology and location of incisors make them more susceptible to TDIs [25].

The prevalence of untreated injures was 93.6%, which is alarming. This shows that treatment of TDIs among schoolchildren is extremely neglected. However, most injuries were enamel fractures, and no treatment was needed for approximately half of untreated TDIs recorded in the present survey. The same result was reported by Hamdan and Rajab [10] who observed that only 3.1 percent of traumatized teeth were treated, and the proportion of teeth needing treatment was smaller than those untreated. Similar results of untreated dental injuries were reported in Palestine [14], Iraq [22] and South Africa [24]. This reflected a low priority of dental health relative to other health problems, and a poor access to dental services. Moreover; the cost of dental treatment may act as a barrier. In agreement with other studies [2,26], falls and collisions were the most common causes of injury. However; a clear universal system to classify causes of injury is still lacking. For example; violence can be misinterpreted as collision, and it is difficult to classify falls during playing sports under falls, sports, or as a result of collisions [27].

Home was the most common place where injury occurred followed by street. These results are similar to those recorded in previous studies [20,22], and emphasize on the role of preventive strategies in providing environments that are favorable to health [25].

As reported in most previous studies, males were more prone to TDIs than females with a ratio of 1.4:1; two previous prevalence studies carried out in Jordan indicated a slightly higher ratio of 1.7:1 [9], and 1.6:1 [11]. Cultural reasons within the Jordanian community make females more conservative and less exposed to vigorous outdoor activities [26,27]. Males are more engaged in contact sports, entertainment games, or fights of a generally more aggressive nature or with a greater risk taking behavior than females do [25]. In disagreement with previous studies [10,11,20,24], more prevalence of TDIs in the present study were found for children from rural areas (19.1%) comparing to urban areas (15.6%), however, no statistically significant relationship was observed. There were no statistically significant differences in the experience of TDIs between children according to the types of schools. Furthermore; it was noticed that the type of school was not a good indicator about the socioeconomic status as some private schools especially in unprivileged areas presented with reduced quality of hygiene measures comparing to other public and UNRWA schools in the same area. Neither parents’ level of education nor family income was significantly associated with the occurrence of TDIs. This was in agreement with most previous studies [4,28,29]. Family income was a vague indicator about socioeconomic status, since parents tend to have a subjective evaluation about their income and some prefer to be conservative. Finding an accurate operational definition of socioeconomic status is still a major problem in most studies and within the Jordanian population.

In agreement with the finding of previous study [30], both inadequate lip coverage and increased overjet were significant associated factors for maxillary incisors trauma (P < 0.05), with inadequate lip coverage the most important (P < 0.001). According to multiple logistic regression analysis, overjet was not a strong predictor for TDIs occurrence as previously assumed [31,32], and lip coverage was the principal predisposing factor for TDIs. This finding supports the results of Burden [17] who concluded that inadequate lip coverage is a better predictor of TDIs than increased overjet. It is assumed that soft tissue coverage from upper and lower lip acts as a protective factor from TDIs even in children with increased overjet. It is the inadequate lip coverage that predisposes children mostly to the traumatic sequel of falls and collisions on teeth. It is worthy to mention that overjet size recorded in the present survey measures only the horizontal distance between upper and lower central incisors. Overjet size does not give an accurate indication about proclination of maxillary central incisors. Small recordings of overjet might be the result of compensatory mandibular incisors proclination, resulting in false interpretation of maxillary incisors protrusion. Further investigations are needed to assess maxillary incisors protrusion through cephalometric radiographs, to uncover if the inclination of maxillary incisor teeth rather than the overjet size contributes to the occurrence of TDIs. It must be borne in mind that this is a cross-sectional study that can be used to explore associations, not causation.

Normal distribution of anthropometric measurements in the sample indicated good representation of the population investigated. Since there is no consensus in the literature to define weight categories in children and adolescents and there is no cut off points for the Jordanian population, the CDC-growth charts specific for age and sex were used to evaluate weight categories for 12-year-old Jordanian schoolchildren, and consequently obesity in the present study. This is an advantage that makes the results of this study comparable to other previous studies using the same methods. However, it might not give an accurate presentation of this medical condition since the life style of the Jordanian population and other developing countries is different from developed countries. This study did not demonstrate a significant association between TDIs to permanent anterior teeth and obesity among 12-year-old schoolchildren in Jordan (P >0.05). This corroborates with the findings of Soriano et al. [6], Ărtun and Al-Azemi [7], and Damé-Teixeira et al. [8]. However; other authors supported the opposite. Petti et al. [3] reported that the probability of dental injury was significantly affected by obesity and suggested that the lifestyle of obese children make them more prone to injuries due to lack of physical skills. Nicolau et al. [4] reported that overweight children were about two times more likely to have dental injuries than other children in Brazil. In the present study, TDIs were more prevalent in non-obese children (16.9%) than in obese ones (10.7%). However, the difference was not statistically significant (P > 0.05). Physical activities might have helped children to obtain healthy weight ranges but at the same time may increase their proneness to TDIs because of the surrounding environment. In Jordan as any developing country, children are engaged in different kind of sports played in unsafe environment without the use of mouth protection. An important methodological argument of the present study is that obesity was not investigated at the time of injury, making the interpretation of data about the association of TDIs and obesity less representative. Other hospital-based studies are needed where anthropometric measurements are assessed at the time of injury. In addition, the possibility that BMI has failed to distinguish between fat and fat-free mass (muscle and bone) may present another shortcoming of the study suggesting that the index could exaggerate obesity in large and muscular boys. Moreover; growth charts specific for the Jordanian population are needed.

## Conclusions

The results of this epidemiological survey show that there is no significant association between TDIs and obesity among 12-year-old Jordanian schoolchildren, and inadequate lip coverage is the principal orofacial risk factor for TDIs. Although common, TDI remains a neglected oral condition since the majority of traumatized teeth remain untreated indicating low priority of dental health among the studied population. Public health promotion programs targeted at populations and high risk groups are highly recommended to reduce the prevalence of TDIs among children.

## Competing interests

The authors declare that they have no competing interests.

## Authors’ contributions

TTA performed the survey and LDR conceived the study and supervised the work. Both authors helped to draft the manuscript. Both authors have read and approved the final manuscript.

## Pre-publication history

The pre-publication history for this paper can be accessed here:

http://www.biomedcentral.com/1472-6831/14/101/prepub
